# Serum Levels of the Cancer-Testis Antigen POTEE and Its Clinical Significance in Non-Small-Cell Lung Cancer

**DOI:** 10.1371/journal.pone.0122792

**Published:** 2015-04-10

**Authors:** Qi Wang, Xuefei Li, Shengxiang Ren, Ningning Cheng, Mingchuan Zhao, Yishi Zhang, Jiayu Li, Weijing Cai, Chao Zhao, Wa Cao, Caicun Zhou

**Affiliations:** Department of Lung Cancer and Immunology, Shanghai Pulmonary Hospital, Tongji University, Tongji University Medical School Cancer Institute, Shanghai, People’s Republic of China; University of North Carolina School of Medicine, UNITED STATES

## Abstract

**Background:**

POTEE (POTE ankyrin domain family, member E) is a newly identified cancer-testis antigen that has been found to be expressed in a wide variety of human cancers including cancers of the colon, prostate, lung, breast, ovary, and pancreas.

**Aim:**

To measure the serum levels of POTEE in patients with non-small-cell lung cancer (NSCLC) and to explore the clinical significance of POTEE in NSCLC.

**Patients and Methods:**

104 NSCLC patients, 66 benign lung disease patients and 80 healthy volunteers were enrolled in this study from May 2013 to February 2014. Serum POTEE levels were measured using enzyme-linked immunosorbent assay (ELISA). Numerical variables were recorded as means ± standard deviation (SD) and analyzed by independent *t* tests. Categorical variables were calculated as rates and were analyzed using a χ^2^ test or Fisher’s exact test. Survival curves were estimated and compared using the Kaplan-Meier method and log-rank tests.

**Results:**

Serum POTEE levels were significantly higher in NSCLC patients than in benign lung disease patients and healthy controls (mean ± SD [pg/ml], 324.38± 13.84 vs. 156.93 ± 17.38 and 139.09 ± 15.80, *P*<0.001) and were significantly correlated with TNM stage. Survival analysis revealed that patients with low serum POTEE had longer progression-free survival (PFS) than those with high serum POTEE (*P*=0.021). Cox multivariate analysis indicated that POTEE was an independent prognostic factor of progression-free survival (*P* =0.009, hazard ratio, 2.440).

**Conclusions:**

Serum POTEE level in NSCLC patients is associated with TNM stage and is a potential prognostic factor.

## Introduction

Lung cancer is the leading cause of cancer-related deaths worldwide, with non-small-cell lung cancer (NSCLC) representing approximately 80% of these cases [1, 2]. Most patients are diagnosed at an advanced stage of disease [3]; they have little prospect of effective and curative treatment. Five-year survival rates are less than 15% [4]. A promising breakthrough with the potential to improve outcomes for NSCLC patients involves the introduction of validated biomarkers into clinical management. These biomarkers may be crucial not only for early diagnosis but also to inform treatment decisions to achieve optimal therapeutic interventions [5, 6].

Cancer testis (CT) antigens are a type of protein that is expressed in normal adult tissue only in the testis as well as a variety of tumors of different histological origins. In view of their pattern of expression and high immunogenicity, CT antigenss are considered very attractive candidates as cancer biomarkers and vaccines [7–9]. POTEE (POTE ankyrin domain family, member E) is a newly identified cancer testis antigen expressed in normal testis, ovary, and placenta, and in many cancers, including those of the prostate, colon, lung, breast, ovary, and pancreas [10–12]. In humans, POTE gene family consists of 13 highly homologous members located on 8 different chromosomes. Previous studies have shown that POTE molecules are localized in the cytoplasm towards the inner aspect of cellular membrane [13]. POTEE, a dominant subtype expressed in many cancers and cancer cell lines [11, 14]. Our previous study revealed the differential expression of POTEE between cisplatin-resistant A549/DDP and its parental A549 cells by proteomics analysis [15].

However, the functions and roles of POTEE in lung cancer remain unclear. In this study, we preliminarily explored the relationship between serum POTEE level and clinical characteristics and survival in NSCLC patients.

## Materials and Methods

### Patient, Healthy Controls and Serum Samples

Peripheral blood was drawn from patients and healthy donors at Shanghai Pulmonary Hospital from May 2013 to February 2014. Sera were obtained from a study cohort of 250 participants as follows: 104 patients with NSCLCs, including adenocarcinoma, squamous cell carcinoma, and large-cell anaplastic carcinoma; 66 benign lung disease patients; and 80 healthy donors. The inclusion criteria for the NSCLC patients were as follows: (1) pathologically confirmed NSCLC; (2) age ≥18 years old; (3) had not received any anti-tumor therapy. Tumor staging was determined according to the 2009 TNM staging classification system. Demographic and pathological data, including age, gender, and smoking history, were collected. The inclusion criteria for benign lung disease patients were as follows: (1) no history of cancer; (2) benign lung disease patients confirmed by pathogen or pathology detection; (3) the diagnosis of benign lung disease patients were bronchiectasis, chronic obstructive pulmonary disease, tuberculosis, pneumonia, chronic bronchitis and lung abscess, the characteristics and demographics of these patients are included in S1 Table; (4) age ≥18 years old. Patients that did not meet aforementioned criteria were excluded. This study was approved by the Ethics Committee of Shanghai Pulmonary Hospital. Written informed consent was obtained from each study participant.

### Enzyme-Linked Immunosorbent Assay

Serum POTEE levels were determined by enzyme-linked immunosorbent assay (ELISA) using an immunoassay kit (Wuhan Huamei Biotech, China; Cat. no.CSB-EL740863 HU) according to the manufacturer’s protocol. The optical density (OD) at 450 nm was measured, and the standard curves were established with OD 450 as the Y axis; these curves were used to determine protein expression levels. Results are reported as the concentration of POTEE (pg/ml) in the serum sample.

### Measurement of common serum tumor markers

Serum tumor markers, including carcinoeyonmbric antigen (CEA), cytokeratin (CYFRA21-1) and carbohydrate antigen 19–9 (CA19-9) were determined by radioimmunoassay (RIA) using CEA, CYFRA21-1(CIS Bio International) and CA19-9 (China Institute of Atomic Energy) RIA kits according to the manufacturer’s protocol. Results were gained by 1470 WIZA RDTM counter instruments.

### Statistical Analysis

Statistical analyses were performed using SPSS 17.0 software (Chicago, IL, USA) and GraphPad Prism 5 (GraphPad Software, San Diego, CA, USA). Numerical variables were recorded as means ± SD and analyzed using independent *t* tests. Categorical variables are presented as rates and were analyzed using χ^2^ test or Fisher’s exact test. To evaluate the diagnostic potential of POTEE, the receiver operating characteristics (ROC) curve was plotted using data produced by logistic regression analysis, and the area under the curve (AUC) was calculated. Survival curves were estimated and compared using the Kaplan-Meier method and log-rank tests. Univariate Cox regression was performed on each clinical covariate to examine its influence on patient survival. Multivariate models were based on step-wise addition. A *P* value <0.05 was considered statistically significant.

## Results

### Demographic Characteristics of All Study Participants

Our study cohort of 250 participants comprised 104 NSCLC patients, 66 benign lung disease patients, and 80 healthy subjects. Mean age was 61.3 ± 9.5 yr in the NSCLC patients group, 58.5 ± 11.5 yr in the benign lung disease patients group, and 57.9 ± 13.6 yr in the healthy control group (*P*>0.05). The basic characteristics of 104 patients with NSCLC, 66 benign lung disease patients, and 80 healthy control subjects are summarized in Table 1.

**Table 1:** Basic Characteristics of Study Participants.

Variables	NSCLC (n, %)	Benign (n, %)	Controls (n, %)	*P* value
Age				0.097
≥65 yr	33 (31.7)	21 (31.8)	26 (32.5)	
<65 yr	71 (68.3)	45 (68.2)	54 (67.5)	
Sex				0.410
Male	59 (56.7)	43 (65.2)	52 (65.0)	
Female	45 (43.3)	23 (34.8)	28 (35.0)	
Smoking status				0.232
Yes	66 (63.5)	34 (51.5)	43 (53.7)	
No	38 (36.5)	32 (48.5)	37 (46.3)	

### Serum POTEE levels in NSCLC Patients, Benign Lung Disease Patients, and Healthy Control Subjects

**Fig 1:**
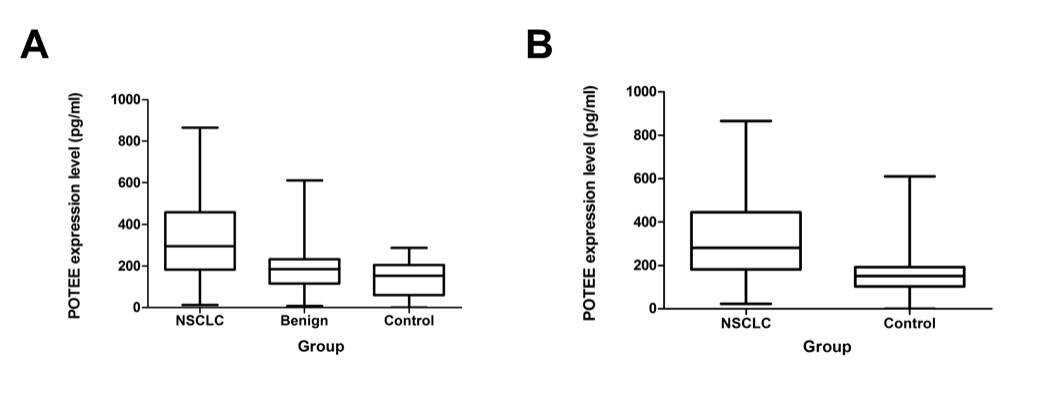
**1A. Serum POTEE levels in NSCLC patients, benign disease and healthy controls.** Mean POTEE level was 324.38 ± 13.84 pg/ml in the NSCLC group, 156.93 ± 17.38 pg/ml in benign lung disease group, and 139.09 ± 15.8 in control group (*P*<0.001). **1B. Serum POTEE levels in patients with NSCLC and controls.** Mean POTEE level was 324.38 ± 13.84 pg/ml in the NSCLC group and 139.09 ± 15.8 pg/ml in control group (*P*<0.001).

Serum POTEE levels were significantly higher in NSCLC patients than in benign lung disease patients and healthy control subjects. The mean serum POTEE level was 324.38 ± 13.84 pg/ml for the NSCLC patient group, 156.93 ± 17.38 pg/ml for the benign lung disease group, and 139.09 ± 15.80 pg/ml for the healthy control group (*P*<0.001) (Fig 1A). The mean serum POTEE level in NSCLC patients was also significantly higher than the mean of 147.15 ± 6.58 pg/ml for all non-NSCLC participants (*P*<0.001) (Fig 1B).

### The Potential of POTEE as a Serologic Tumor Biomarker in NSCLC

**Fig 2:**
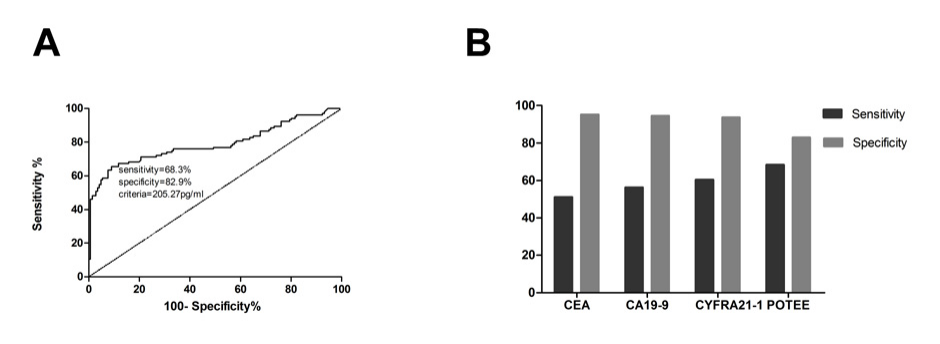
**2A. ROC curve of POTEE as a serologic tumor marker in NSCLC.** Using a cut off value 205.27 pg/ml, ROC analysis revealed an area under curve (AUC) of 0.793 (*P*<0.01), indicating a high sensitivity and specificity to differentiate benign lung disease group and healthy controls from NSCLC patients. **Fig 2B. Diagnostic values of CEA, CYFRA 21–1, CA199 and POTEE in N SCLC.** CEA, CYEFRA 21–1, CA199 and POTEE sensitivity were 51.0%, 56.3%, 60.4% and 68.3%, the specificity were 95.2%, 94.5%, 93.8% and 82.9%.

A ROC curve analysis was carried out to assess the value of POTEE in NSCLC diagnosis. ROC curve is to reflect the sensitivity and specificity of continuous variable comprehensive index, the area under the curve the greater the diagnosis accuracy is higher. The AUC was 0.793 (95% confidence interval [CI], 0.7–0.9). We chose the point with highest sensitivity and specificity, 205.27 pg/ml, as the cutoff value. With the cutoff value, which was defined as the normal value based on the mean value (plus two standard deviations) obtained from healthy controls, serum POTEE achieved a diagnostic sensitivity of 68.3% and a specificity of 82.9% (Fig 2A). We also detected the serum levels of commonly used serologic tumor markers (TM), including carcinoeyonmbric antigen (CEA), carbohydrate antigen 19–9 (CA19-9), and cytokeratin (CYFRA21-1) in the NSCLC group. Their sensitivities were 51.0% (CEA), 56.3% (CA19-9), and 60.4% (CYFRA21-1), the specificity was 95.2% (CEA), 94.5% (CA19-9), and 93.8% (CYFRA21-1), and the accuracy was 77.7% (CEA), 79.3% (CA19-9), and 80.6% (CYFRA21-1) (Fig 2B). Among the 104 NSCLC patients, the combined detection sensitivity of CEA, CA19-9, CYFRA21-1, and POTEE was 89.3%, which was obviously higher than the sensitivity of any single detection (*P*<0.05).

### Relation of Serum POTEE to Clinicopathological Characteristics

As shown in Table 2, serum POTEE level was significantly correlated with TNM stage. Patients in advanced TNM stages had higher POTEE levels than those with early stages of lung cancer (*P* = 0.036). However, no significant correlation was observed between serum POTEE level and other clinicopathologic parameters, including age, gender, smoking history, pathology, or family history.

**Table 2:** Relation of Serum POTEE to Clinicopathological Characteristics of 104 Patients with NSCLC.

Group	Categories	n (%)	POTEE levels (pg/ml)	*P* value
Gender	Male	59 (56.7)	327.30 ± 25.83	0.864
	Female	45 (43.3)	320.55 ± 29.58	
Age, yr	≥65	33 (31.7)	311.64 ± 34.51	0.656
	<65	71 (68.3)	330.30 ± 23.53	
Histological type	Adenocarcinoma	64 (61.5)	294.03 ± 25.02	0.907
	Squamous	29 (27.9)	312.62 ± 37.17	
	Adenosquamous	11 (10.6)	309.67 ± 60.35	
TNM stage^#^	I–IIIA	25 (24.0)	252.60 ± 38.85	0.036*
	IIIB–IV	79 (76.0)	347.09 ± 21.85	
PS score	0–1	94 (90.4)	313.13 ± 20.54	0.228
	2	10 (9.6)	229.85 ± 59.75	
Family history	Yes	11 (10.6)	262.06 ± 80.08	0.495
	No	93 (89.4)	305.45 ± 19.73	
Smoking history	Yes	66 (63.5)	338.63 ± 28.19	0.142
	Never	38 (36.5)	279.12 ± 25.78	

PS, performance status;

^#^, 2 patients in stage I, 5 patients in stage II, 18 patients in stage IIIA, 34 patients in stage IIIB, 45 patients in stage IV;

*, serum POTEE level was associated with TNM stage.

### Association Between Serum POTEE Level and Efficacy of Platinum-Based Chemotherapy

We analyzed whether serum POTEE level is related to tumor response to chemotherapy. Among the 104 patients with NSCLC, 76 patients were at TNM stage IIIb–IV and received doublet chemotherapy. Among 76 patients, 33 patients had been treated with gemcitabine plus cisplatin, 23 had been treated with vinorelbine plus cisplatin, 11 had been treated with paclitaxel plus cisplatin and 9 had been treated with vinorelbine plus carboplatin. The POTEE serum level cutoff value of 205.27 pg/ml with highest sensitivity and specificity was selected to categorize patients as high POTEE (n = 43) and low POTEE (n = 33) groups. We analyzed patient responses to platinum-based chemotherapy (Table 3). For patients with high POTEE expression (≥205.27 pg/ml), objective response rate (ORR) and disease control rate (DCR) were significantly lower than for those with low POTEE expression (<205.27 pg/ml) (*P* = 0.023; *P* = 0.043). Serum POTEE expression was strongly negatively correlated with the response of patients to platinum-based chemotherapy.

**Table 3:** Association Between Serum POTEE Level and Efficacy.

	High POTEE expression (≥205.27 pg/ml)	Low POTEE expression (<205.27 pg/ml)	*P* value
PR, n	7	13	
SD, n	12	9	
PD, n	24	11	
ORR, %	16.3	39.3	0.023
DCR, %	44.2	66.6	0.043

ORR, objective response rate; DCR, disease control rate; PR, partial response; PD, progression disease; SD, stable disease.

### Association between Serum POTEE Level and Survival

**Fig 3:**
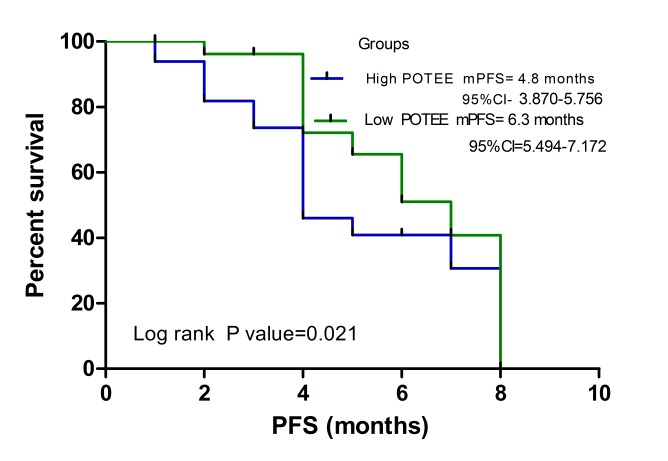
Kaplan-Meier survival curves in relation to serum POTEE level in patients with NSCLC. Survival curves were analyzed by Kaplan-Meier method and log-rank test. Patients with high POTEE levels had a significantly poorer survival than those with low POTEE levels (*P* = 0.021).

In the Kaplan-Meier survival curve analysis, the group of patients with low POTEE expression had a significantly longer median progression-free survival (PFS; 6.3 months; 95% CI, 5.494–7.172) than the group with high POTEE expression (PFS, 4.8 months; 95% CI, 3.870–5.756) (*P* = 0.021) (Fig 3). As shown in Table 4, a Cox proportional hazards analysis was used to further evaluate the potential of serum POTEE expression level as a prognostic biomarker. Univariate analysis showed that serum POTEE expression level was associated with prognosis (HR, 1.913; *P* = 0.040). In the multivariate Cox proportional hazards analysis, low POTEE expression level was significantly associated with longer PFS (HR, 2.440; *P* = 0.009). As expected, disease stage was strongly associated with shorter PFS in both univariate and multivariate analyses (*P*<0.05).

**Table 4:** Univariate and Multivariate Analyses of POTEE Status with Regard to PFS.

	Univariate analysis			Multivariate analysis		
Variables	HR	95% CI	*P* value	HR	95% CI	*P* value
POTEE level (low vs. high)	1.913	1.030–3.551	0.040*	2.440	1.252–4.757	0.009*
Age (≥60 vs. <60, yr)	1.171	0.653–2.097	0.597	1.168	0.633–2.154	0.620
Sex (male vs. female)	0.728	0.399–1.328	0.301	0.591	0.314–1.113	0.103
Histology	1.115	0.753–1.652	0.586	1.140	0.722–1.801	0.573
TNM stage (I-IIIA vs. IIIB-IV)	4.089	1.818–9.199	0.001*	4.789	2.082–11.014	0.001*
Smoking history	1.321	0.720–2.424	0.369	0.807	0.366–2.776	0.593
Family history	0.557	0.172–1.798	0.328	1.927	0.504–7.376	0.338

CI, confidence interval; HR, hazard ratio;

*, PFS was associated with POTEE level and TNM stage.

## Discussion

To our knowledge, this study is the first to assess POTEE expression in serum by ELISA to examine its clinical significance in NSCLC. We observed that the expression of POTEE in sera was significantly higher in NSCLC patients compared with benign lung disease patients and healthy volunteers. Furthermore, we preliminarily analyzed the clinical significance of POTEE in NSCLC patients. Levels of serum POTEE were significantly correlated with TNM stage. Survival analysis revealed that patients with low serum POTEE levels had longer progression-free survival (PFS) than those with high POTEE levels. High POTEE expression levels were associated with poor response to chemotherapy in NSCLC patients. Moreover, multivariate analysis also showed that high POTEE expression was an independent factor for poor survival in NSCLC patients. These results suggest that POTEE expression may be associated with prognosis of NSCLC patients and could serve as a potential biomarker.

Up to date, few previous studies reported an association between POTEE and cancer prognosis because POTEE is a newly identified CT antigen. The gene family POTE is classified into three groups: group 1 includes only POTE 8; group 2 includes POTE15, -18, and -21; and group 3 includes POTE2, -14, and -22 [16, 17]. In the initial studies, POTE proteins were thought to be located at the inner plasma membrane and were classified as CT antigens because they are expressed in many cancers and some normal reproductive tissues [16, 17]. Several previous studies have reported POTE roles in both physiologic and pathologic conditions, including the cytoskeletal structure [8], apoptotic pathways [11], epigenetic regulation of tumor progression [18], and lineage-specific differentiation of embryonic cells [19]. However, the function of POTE in cancer remains unknown. Refield et al. studied the subcellular distribution of all POTE proteins and their association with the progression and metastasis of malignancies. They found that POTE proteins correlated well with malignant progression and metastasis in a variety of tissues and that POTEG and /or POTEH are pivotal for cancer cells to grow and survive [20]. Liu et al. found that transient expression of POTEE or POTEF induces apoptosis in Hela cells. These findings indicate that the POTE gene family encodes a family of pro-apoptotic proteins [21]. Yi-Hsiang Huang et al. determined peptide modification can enhance the immunogenicity of POTE epitopes to induce T cells that kill human cancer cells, this result suggest that POTE may also be a molecular target for cancer immunotherapy [22].

In the last few years the researches for CT antigen have made some progress. The unique expression pattern of CT antigen makes them become candidates for use in early diagnosis and as a prognostic marker. The CT antigen MAGE has been found in 85% of NSCLC cases, 75% of early multiple myeloma cases, and 35% early breast cancer cases [23]. The CT antigen NY-ESO-1 has also been identified in 32% of early NSCLC cases, 29% of esophageal cancer cases, and 23% of early transitional cell carcinoma case [23]. These CT antigens can be used as biomarkers for early diagnosis of tumors. Importantly, MAGE can be detected in the peripheral blood of hepotocellular carcinoma patients, and their expression levels are closely related to metastasis status and prognosis in hepatocellular carcinoma patients [24–26]. In addition, MAGE, BAGE and GAGE CT antigen can be detected in the ascites of patients with ovarian cancer and in the cerebrospinal fluid of patients with brain metastases from melanoma. Thus, CT antigens can be used as biomarkers of tumor metastasis and prognosis [27, 28]. Because of their pattern of expression, CT antigens are very attractive candidates for immune-based therapies of cancer, and many clinical trials are testing their use in cancer patients [9, 29, 30].

In the present study, there had some limits. Our study cohort size was small; to establish the clinical utility of POTEE in serum, further studies should be performed with larger cohorts. In order to improve the specificity of POTEE ROC curve, we consider the following several aspect work needs to be done: (1) to enlarge the sample size to further optimize the cutoff value and confirm our results; (2) further, the perspective study should be performed; (3) to try the available combination of serum tumor biomarkers. In addition, we also need to investigate the functional mechanisms of POTEE in NSCLC.

In conclusion, our results show that POTEE is over-expressed in NSCLC and that POTEE level in serum is associated with TNM stage and prognosis in NSCLC patients.

## Supporting Information

S1 Table Characteristics and Demographics of Benign Lung Disease Patients. (DOC)
