# Supplementary material for: Serum Levels of the Cancer-Testis Antigen POTEE and Its Clinical Significance in Non-Small-Cell Lung Cancer
Source: PLoS One. 2015 Apr 10;10(4):e0122792. doi: 10.1371/journal.pone.0122792 (PMC4393100; doi:10.1371/journal.pone.0122792)
Supplement: S1 Table — (DOC) [file pone.0122792.s001.doc]

**S1 Table . Characteristics and Demographics of Benign Lung Disease Patients**.

| Group | Categories | n (%) | Severity (n) |
| --- | --- | --- | --- |
| Gender | Male | 43 (65.2) |  |
|  | Female | 23 (34.8) |  |
| Age, yr | ≥65 | 21 (31.8) |  |
|  | <65 | 45 (68.2) |  |
| Disease Type | Bronchiectasis | 13(19.7) |  |
|  | COPD | 16(24.2) | I(3), II(6), III(4), IV(3) |
|  | Tuberculosis | 13(19.7) |  |
|  | Pneumonia | 9(13.6) |  |
|  | Chronic bronchitis | 12(18.2) |  |
|  | Lung abscess | 3(4.5) |  |

COPD, chronic obstructive pulmonary disease.
